# Development of a novel drug information provision system for Kampo medicine using natural language processing technology

**DOI:** 10.1186/s12911-023-02230-3

**Published:** 2023-07-13

**Authors:** Ayako Maeda-Minami, Tetsuhiro Yoshino, Tetsuro Yumoto, Kayoko Sato, Atsunobu Sagara, Kenjiro Inaba, Hidenori Kominato, Takao Kimura, Tetsuya Takishita, Gen Watanabe, Tomonori Nakamura, Yasunari Mano, Yuko Horiba, Kenji Watanabe, Junzo Kamei

**Affiliations:** 1 Faculty of Pharmaceutical Sciences, Tokyo University of Science, Noda, Yamazaki, Chiba 2641 Japan; 2 Center for Kampo Medicine, Keio University School of Medicine, 35, Shinanomachi, Shinjuku-ku, Tokyo, Japan; 3 Hoshi University, 2-4-41 Ebara, Shinagawa-ku, Tokyo, Japan; 4 Department of Pharmacy, General Sagami Kosei Hospital, Oyama, Chuou-ku, Sagami, Kanagawa 3429 Japan; 5 I&H Co. Ltd, 1-18 Oomasuchou, Ashiya, Hyogo Japan; 6 Kimura Information Technology Co. Ltd, 6-1 Oroshihonmachi, Saga, Saga Japan; 7 Division of Pharmaceutical Care Sciences, Center for Social Pharmacy and Pharmaceutical Care Science, Faculty of Pharmacy, Keio University, 1-5-30, Shibakoen, Minato-ku, Tokyo, Japan; 8 Juntendo Advanced Research Institute for Health Science, Juntendo University, 2-1-1, Hongou, Bunkyo-ku, Tokyo, Japan

**Keywords:** Natural language processing, Drug information provision system, Kampo medicine, Conversational agents, Chatbots, Question-answering

## Abstract

**Background:**

Kampo medicine is widely used in Japan; however, most physicians and pharmacists have insufficient knowledge and experience in it. Although a chatbot-style system using machine learning and natural language processing has been used in some clinical settings and proven useful, the system developed specifically for the Japanese language using this method has not been validated by research. The purpose of this study is to develop a novel drug information provision system for Kampo medicines using a natural language classifier® (NLC®) based on IBM Watson.

**Methods:**

The target Kampo formulas were 33 formulas listed in the 17th revision of the Japanese Pharmacopoeia. The information included in the system comes from the package inserts of Kampo medicines, Manuals for Management of Individual Serious Adverse Drug Reactions, and data on off-label usage. The system developed in this study classifies questions about the drug information of Kampo formulas input by natural language into preset questions and outputs preset answers for the questions. The system uses morphological analysis, synonym conversion by thesaurus, and NLC®. We fine-tuned the information registered into NLC® and increased the thesaurus. To validate the system, 900 validation questions were provided by six pharmacists who were classified into high or low levels of knowledge and experience of Kampo medicines and three pharmacy students.

**Results:**

The precision, recall, and F-measure of the system performance were 0.986, 0.915, and 0.949, respectively. The results were stable even with differences in the amount of expertise of the question authors.

**Conclusions:**

We developed a system using natural language classification that can give appropriate answers to most of the validation questions.

**Supplementary Information:**

The online version contains supplementary material available at 10.1186/s12911-023-02230-3.

## Background

The widespread use of the Internet has enabled instant access to vast amounts of information related to medications; however, it is necessary to assess the correctness of the information that is obtained [1]. In addition, the aging of the population has increased the use of multiple medications, which is making drug therapy more complex and increasing the risk of adverse reactions and drug interactions [1–3]. There is a need for a drug information provision system that enables people to obtain reliable drug information quickly.

In Japan, traditional Kampo medicines (漢方薬) are widely used [4–7]. Traditional Kampo medicines, broadly similar to traditional Chinese medicines but differing from them in some important aspects, were approved by the national health insurance system in the late 1960s and have been widely used in Japan until the present day. The theoretical basis of these medicines is found in Shanghan-lun and Jingui Yaolue; however, clinical trials over the last three to four decades have caused practitioners of data-based empirical medicine to take them seriously [8]. Kampo medicines employ combinations of several crude drugs, mostly derived from herbs, and their proper usage is important [9, 10]. Currently, there is only one type of physicians’ license in Japan; therefore, every physician can prescribe traditional Kampo medicine. This contrasts with the situation in other countries where traditional Chinese medicine is used. More than 90% of physicians prescribe Kampo medicines [11]. The same is true of pharmacists, and more than 80% of medical facilities (such as hospitals and pharmacies) dispense Kampo medicines [12]. However, 70% of physicians have never studied Kampo medicine [5] and even pharmacists felt that they had insufficient knowledge of the indications, off-label use, adverse reactions, and combinations of Kampo medicines [13]. In fact, one in four prescriptions of Kampo medication administered to elderly patients was potentially inappropriate [13]. Therefore, the potential exists for adverse outcomes from the use of Kampo medicines.

Centers for drug information, where experts are available to answer questions, have been established in each country to help healthcare providers obtain reliable drug information [1, 14, 15]. In addition, applications on mobile devices may enable healthcare providers to obtain drug information more easily and quickly [15–17]. One technology for quickly providing evidence-based, official, and public information is a chatbot-style system using machine learning and natural language processing (NLP) [18–20]. Once a user asks a question in natural language, the system takes the intention of the question and returns a predefined answer. The development of a system using this method has the following two advantages. First, inexperienced medical professionals do not need to judge the evidence of the information obtained from the system. Second, even inexperienced health care providers can use natural language to ask questions in a conversational manner, which allows them to retrieve more information than from the traditional methods, such as reading textbooks or making phone calls [19]. The literature reports that this method has been used in various chatbots, including systems where healthcare workers could search for information on Micromedex [19, 20] and experts from government agencies and a health plan delivered information related to coronavirus disease 2019 (COVID-19) to the public [21]. IBM Watson’s Natural language classifier® (NLC®, currently renamed Assistance®) performs natural language classification [18–21] as a chatbot-style system; this and other NLP systems have proved useful in some clinical settings [19–22]. Although there are several systems for the Japanese language, none have been studied for research purposes.

The purpose of this study is to develop a drug information provision system for Kampo medicines using an NLC® based on IBM Watson. The system developed in this study is a prototype built on limited basic information, and it is highly versatile in actual clinical practice. The performance of this system was evaluated with 900 validation questions provided by six pharmacists (classified into high or low levels of knowledge and experience with Kampo medicines) and three pharmacy students.

## Methods

### Data source

The target Kampo formulas were 33 prescriptions listed in the 17th revision of the Japanese Pharmacopoeia [23]. The information in the present system was obtained from the package inserts of Kampo medicines, the Manuals for Management of Individual Serious Adverse Drug Reactions (hereinafter referred to as “the Manuals”), and information on off-label use [12]. The package inserts and the Manuals are the official sources of information in Japan. The package inserts are made by the manufacturers under the provisions of the Pharmaceutical Affairs Law to provide necessary information to physicians, dentists, pharmacists, and other medical personnel to ensure the safety of patients. The package inserts are considered to be the only documents with legal basis; they include contraindications, description (composition and properties), indications, dosage and administration, precautions, careful administration, important precautions, drug interactions, adverse reactions, use in the elderly and pediatric populations, use during pregnancy, delivery or lactation, and pharmacology [23]. The indications listed in the package inserts are approved by the Ministry of Health, Labor, and Welfare and are covered by insurance in Japan when the drug is used for the diseases listed in the indications. The Manuals were made by a committee commissioned by the Ministry of Health, Labor, and Welfare [24]. The Manuals discuss subjective symptoms; physical, imaging, and pathological findings; mechanism, frequency, and timing of occurrence; identification criteria; diseases requiring identification; identification methods; monitoring items; treatment methods; and risk factors for each serious adverse drug reaction [24]. The off-label use information consists of diseases for which each drug is used intentionally by medical practitioners but that are not listed on the package insert as labeled indications. Because there is no official document for off-label use information, we employed the off-label use information in the chapter on Kampo medicine in “Konnichi-no Chiryouyaku,” a book widely used in Japanese medical institutions and written by the co-authors [25].

### System design

**Fig. 1:**
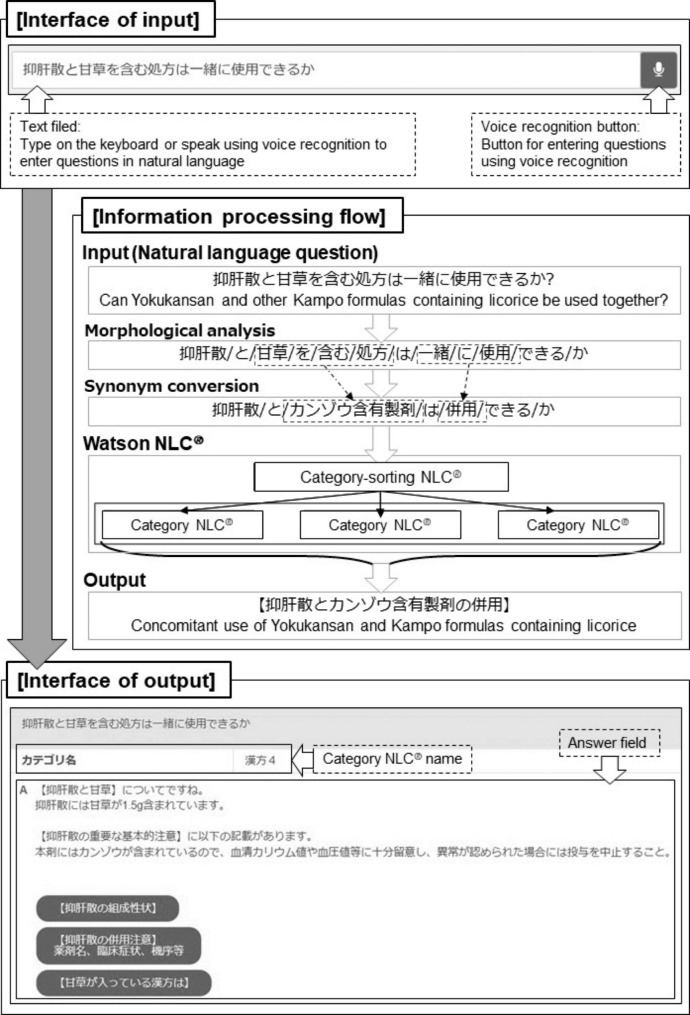
System interface and information processing flow The user can enter a natural language question directly in the text field or press the voice recognition button and speak the question using voice recognition. The input question is first analyzed by morphological analysis and then converted to synonyms based on the thesaurus. Then, Watson’s NLC has two steps: The first step, “Category-sorting NLC,” selects the category that best matches the entered question among six categories. After synonym conversion, the questions are re-entered in the “Category NLC” determined in the “Category-sorting NLC” to get an answer

The system developed in this study classifies natural-language input questions about the drug information of Kampo formulas into preset questions and then outputs the respective preset answers for these questions (Fig. 1). For developing the system, Ruby was used as the programming language because of its high efficiency, known from previous system-development experience. Morphological analysis was performed using MeCab [26], selected because of its rich dictionaries, accuracy, and processing speed after comparing Japanese language libraries at the beginning of development. We used Natural Language Classifier® (NLC®), one of the application programming interfaces of IBM Watson®, which uses NLP as a methodology [18]. The question–answer (QA) set and thesaurus registered in Watson were created using Microsoft Excel [27]; Microsoft OneDrive was used as a collaboration tool [28]. The system procedure was as follows: first, morphological analysis was performed on the input question, and then synonym conversion was performed for words in the thesaurus. Next, input data after synonym conversion were entered into the NLC®, and the input data were classified into classifiers with similar pre-trained sentences. Finally, the defined answers were output.

### System development and optimization

#### System using Natural Language Classifier® (NLC®)

NLC® is IBM’s application programming interface that applies deep learning to NLP to understand the intent of input questions and classify them to predefined answers [18]. The classifier is trained on the defined answers to the training data questions, and it classifies newly given unknown questions to similar training data questions.

The following two steps were necessary in developing the system:

1. We created and registered answers and corresponding training data questions to the NLC® based on the data from the information sources using the following procedure: Before creating the questions, we divided information on the package inserts and the Manuals into 13 and 18 sections, respectively, and used the information written there as the answers. We instructed the QA authors to prepare three types of questions: questions in which the name of the answer section was directly included in the question (e.g., “I want to know the *indications* of Kakkonto (ge-gen-tang; 葛根湯)”, “I want to know the *treatment* of pseudohyperaldosteronism”); questions in which the name of the answer section was not directly included (e.g., “Can I use Kakkonto for stiff shoulders?”, “If pseudoaldosteronism develops, can I use antihypertensive medication?”); and questions that required the system to refer to one section of multiple medications throughout (e.g., “Which Kampo medicines are indicated for rhinitis?”, “Which medications should not be administered to a pregnant woman?”). The total number of answers in the whole NLC® was 2,587.
2. For each registered answer, multiple questions were registered. The total number of questions was 21,786. The minimum number of questions corresponding to each answer required by the system was three because multiple questions per answer increase the likelihood that NLC® will find similar text when searching for an answer. The number of questions corresponding to each answer was seven on median, with three and 165 being the minimum and maximum, respectively. The number of questions corresponding to each answer about indication and adverse reactions tended to be high, especially for drugs with multiple indications and adverse reactions. The expression of questions was composed based on the package insert, the Manuals, and a book in Japanese regarding patients’ subjective descriptions of side effects [29]. The registered answers and corresponding questions were prepared by three pharmacists (including ones certified in Kampo medicine with the special training system) and seven pharmacy students. All questions and answers were reviewed by the pharmacists, and discrepancies were resolved through discussion.

In Watson’s NLC®, the upper limits of the number of answers and corresponding questions in one NLC® are 3,000 and 20,000, respectively, which limits the information that can be put in one NLC®. For the system to provide a variety of information, it needs to contain many answers and corresponding questions and the information needs to be divided into several NLCs®. This means that the user needs to select the NLC® to ask a question in advance. If the user does not know which NLC® should be selected, the correct answer can never be obtained. Therefore, we placed one upstream NLC® (Category-sorting NLC®) and six downstream NLCs® (Category NLC®) (Fig. 1). Users can input their questions even if they do not know which category NLC® should be selected: the sorting NLC® selects the appropriate downstream category NLC®. We selected one representative question among the multiple corresponding ones for each answer in category NLC®; these representative questions were used as questions for the category-sorting NLC®. The answer registered by the category-sorting NLC® was the name of the downstream category NLC® into which the user’s question was then put. As each category NLC® registered multiple questions for each answer, it was possible for these classifiers to present more detailed answers than did the category-sorting NLC®. Because of the limited number of QAs that can be registered in one NLC, we prepared six downstream category NLC®s: four for questions related to package inserts, one for the Manuals, and one for questions that require the system to refer to one section of multiple medications throughout. When a user wants to obtain information derived from one of their previously asked questions, they need not enter the question upstream again: they can move from the NLC® where they once asked the question to another NLC® by clicking on a button to select the desired information.

The NLC® calculates the confidence rate in the answer to the input question. The calculation method of the confidence rate is not disclosed, but it considers the similarity between the input question and the registered question. It is designed such that the sum of all the confidence rates of the candidate’s answers will total to 100%. Our system allows setting a threshold value of a confidence rate. The system answers “I do not have an answer” if the confidence rate is below the threshold value. When users input a question for which no question is registered, our system cannot give a correct answer and outputs a wrong answer. This is because the system outputs the registered answer with the highest confidence rate. If a user asks a question that has a registered answer in the system, it will find a registered question with a high degree of similarity that will likely have a higher confidence rate. In contrast, if a user question has no registered answer, the likelihood of finding a registered question with a high degree of similarity is low and the confidence rate is likely to be low. When the system receives user questions for which no registered answers have been input and the confidence rate is below a certain threshold level, having the system say “I do not have an answer” decreases the likelihood of wrong answers being output.

### Thesaurus

In system development using NLC®, it is time-consuming to increase the number of registered questions corresponding to one answer. Registered questions are created by humans, so the more data the system has, the more time-consuming this becomes. To solve this problem, we created a thesaurus. Fluctuations in natural language expressions can be absorbed by NLC®. However, if we create a system using only NLC®, questions with many synonyms need to be registered, the number of questions will be huge, and individual confidence rates will decrease. Therefore, we decided to perform synonym conversion before questions are input to the NLC®. In the thesaurus, 369 keywords corresponded to 1,429 synonyms. The system used the thesaurus to convert the words in the user questions into keywords. The thesaurus was created in the same way as the questions and answers registered with NLC®. It was constructed by three pharmacists (including ones certified in Kampo medicine with the special training system) and seven pharmacy students. First, we extracted words from the package inserts and the Manuals. Colloquial expressions describing symptoms were extracted from a book in Japanese regarding patients’ subjective descriptions of side effects [29]. Afterward, pharmacists and pharmacy students checked these and added other colloquial expressions to the thesaurus. The thesaurus was reviewed by the pharmacists, and discrepancies were resolved through discussion.

For the system to perform optimally, the answers to the questions entered into the system must match the answers given by the experts after reading the questions. To ensure that the system was optimized, we created multiple questions that might be asked in clinical settings and input them to check whether the system found the correct answers. If it did not, we rechecked the questions in the NLC® and the synonym dictionary. In addition, the developers discussed why the correct answer could not be derived. We modified the words of the registered questions in NLC® and expanded the thesaurus several times by increasing the number of phrasings of questions in the NLC®, registering words used in the questions to the synonym dictionary, and registering words in the thesaurus as one of the wordings of the questions in the NLC®.

### Validation

Validation was conducted to investigate the performance of the system. The 900 validation questions were based on what pharmacists may want to know about using Kampo medicine in a clinical setting, including indications, adverse reactions, and drug interactions. Nine additional people (pharmacists and pharmacy students) who had not participated in any of the previous steps prepared 100 validation questions each. To ensure that the performance of the system was not biased by the pharmacists’ knowledge and experience of Kampo medicines, the nine question-authors were divided based on their knowledge into three groups of three people each: (1) pharmacists with advanced knowledge and experience of Kampo medicines (Advanced group); (2) pharmacists with basic knowledge and experience of Kampo medicines (Basic group); and (3) pharmacy students after practical clinical training based on previous studies [12]. The pharmacists in the first group were certified in Kampo medicines, had experience in dispensing the crude drugs used in Kampo medicines, and had dispensed at least 51 Kampo extracts over at least five years. The second group consisted of pharmacists who did not qualify for membership in the advanced group [12]. The 900 validation questions covered 34.7% of registered answers in our system. The objective “drugs” in the validation questions included five Kampo formulas, Daikenchuto (Da-Jian-Zhong-Tang; 大建中湯), Yokukansan (Yi-gan-san; 抑肝散), Hochuekkito (Bu-Zong-Yi-Qi-Tang; 補中益気湯), Rikkunshito (Liu-Jun‐Zi‐Tang; 六君子湯), and Shakuyakukanzoto (Shao-Yao-Gan-Cao-Tang; 芍薬甘草湯), which are among the ten most-prescribed Kampo formulas in Japan and have a large number of clinical trial registrations and studies [30, 31]. The objective “serious adverse reactions” in validation questions included pseudoaldosteronism and interstitial pneumonia, which are well-known serious adverse reactions of Kampo medicines. The validation questions were created for each objective formula and serious adverse reaction, regardless of whether or not the contents of validation questions were listed in the package insert or the Manuals.

To confirm the threshold of the confidence rate of the system, we drew a receiver operating characteristic (ROC) curve using a threshold. The threshold value at which the value of “Sensitivity-(1-Specificity)” is maximized was obtained. The system was set to answer “I do not have an answer” if the confidence rate was less than this threshold. Additionally, four thresholds with confidence rates of 50%, 60%, 70%, and 61%, derived from the ROC curve analysis, were considered.

The validation questions were classified by the administrator based on whether the question had a correct answer in the system. The questions were put into the system and the confidence rate and answers (“correct”, “incorrect”, or “I do not have an answer”) were recorded. After all 900 questions were input, the precision, recall, and F-measure were calculated for all validation questions and each group.

The true positive (TP), false negative (FN), false positive (FP), and true negative (TN) were calculated as follows: TP was defined as the number of questions for which there was a correct answer in the system and the system gave the correct answer. FN was defined as the number of questions for which there was a correct answer in the system, but the system could not give the correct answer or replied with “I do not have an answer.“ FP was defined as the number of questions that had no correct answer in the system and the system should answer “I do not have an answer” but the system gave some wrong answer. TN was defined as the number of questions for which there was no correct answer in the system and the system was able to answer “I do not have an answer.”

Precision is the quotient of TP divided by the sum of the TP and FP. Recall is the quotient of TP divided by the sum of TP and FN. The F-measure is the harmonic mean of precision and recall.

## Results

The system was completed in July 2021.

Table 1 shows examples of the validation questions, whether answers were in the system, the question author’s answer, answers given by the system, and the judgment on the system answers.

**Table 1:** Examples of the validation questions, answers output by the system, and results

Examples of validation question (Original text was in Japanese)	Answer in system	Question-author’s answers	System answers^a^		System confidence	Judgment	
Can I use Daikenchuto for patients with high AST and ALT? 大建中湯はAST,ALTが高い人に使ってもいいですか?	Yes	Daikenchuto and liver dysfunction	Daikenchuto and liver dysfunction	〇	95.2%	Correct	TP
In what patients is Shakuyakukanzoto contraindicated? 芍薬甘草湯の禁忌はどんな患者さんですか?	Yes	Contraindications to Shakuyakukanzoto	Indication of Shakuyakukanzoto	×	71.4%	Incorrect	FN
Can I give Yokukansan to patients with gastrointestinal upset? 胃腸の調子が良くない患者さんに抑肝散を使ってもいいですか?	Yes	Careful administration of Yokukansan	I do not have an answer.^c^	△	45.6%	Incorrect	FN^b^
Please tell me the crude drug that has a diuretic effect in Rikkunshito. 六君子湯の中で利水利尿作用のある生薬を教えてください。	No	-	I do not have an answer.^c^	△	40.5%	Correct	TN^b^
How many grams of licorice can I take in a day when I take Yokukansan? 抑肝散の一日の甘草の量は何グラムまでならよいですか?	No	-	Yokukansan package	×	95.5%	Incorrect	FP

^a^ 〇 means the results of the system were expected. △ means results of the system were “I do not have an answer.“ × means results of the system were unexpected

^b^When the threshold of confidence rate is set to 61%

TP True positive; FN False negative; TN True negative; FN False negative

If there is an answer in the system to the question (Yes) and the system answer is the expected one (〇), the system answer is judged as correct (TP). If there is an answer in the system to the question (Yes) but the system answer is unexpected (×) or “I do not have an answer” (△), the result is judged as incorrect (FN). If there is no answer to the question in the system (No) and the system answer is “I do not have an answer” (△), the result is judged as correct (TN). If there is no answer in the system to the question (No) but the system gives another one (×), the result is judged as incorrect (FP).

**Fig. 2:**
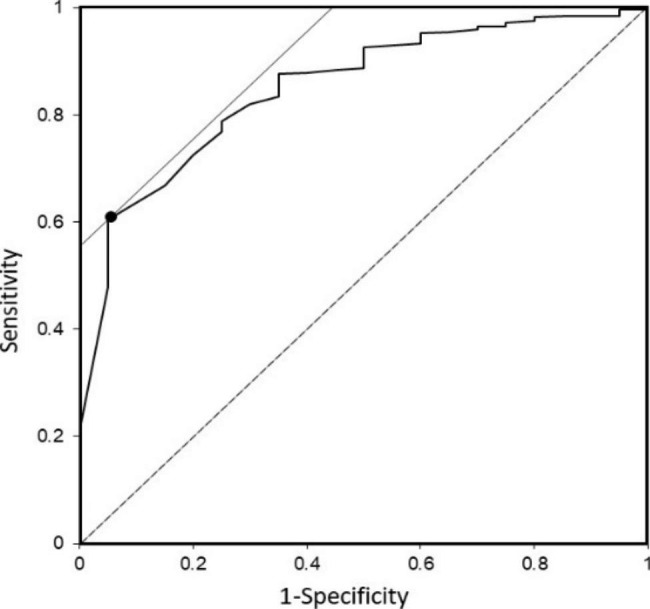
Receiver operating characteristic (ROC) curve using the threshold of confidence rate The best threshold of confidence rate was 61% according to the ROC curve. Precision, recall, and F-measure at the threshold were 0.986, 0.915, and 0.949, respectively. The developed system showed high performance with a precision, recall, and F-measure of approximately 0.9

**Fig. 3:**
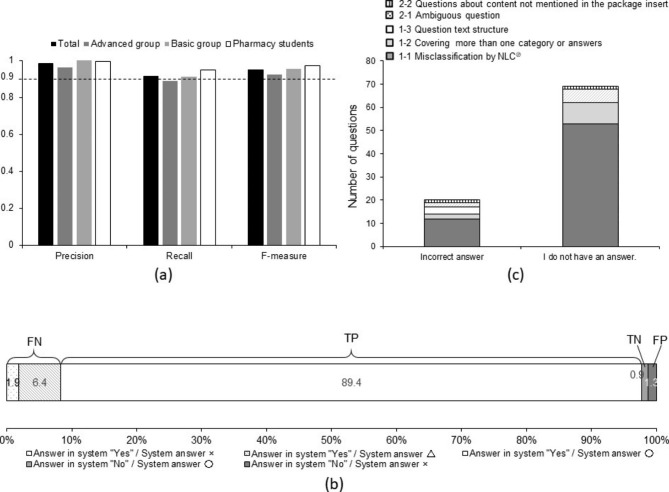
The results of the system by the threshold of confidence rate (**a**) Precision, recall, and F-measure do not vary with the expertise of the question authors. Advanced group: Pharmacists with advanced knowledge and experience of Kampo medicines; Basic group: Pharmacists with basic knowledge and experience of Kampo medicines (**b**) The system was able to provide the correct answer for 90.3% (89.4% for true positive plus 0.9% for true negative) of the validation questions. 〇 means the system answers were expected. △ means the system answers were “I do not have an answer.” × means the system answers were unexpected. TP: True positive; FN: False negative; TN: True negative; FN: False negative (**c**) Number of questions with correct answers listed in the system by the threshold of confidence rate of why the question did not lead to the correct answer. The main reason why questions did not lead to the correct answer even though the correct answer was listed in the system is the Misclassification of Category-sorting NLC® and Category NLC®

To confirm the threshold of confidence rate, we drew an ROC curve (Fig. 2). The best threshold of confidence rate was 61% according to the ROC curve. The precision, recall, and F-measure at the threshold were 0.986, 0.915, and 0.949, respectively (Fig. 3). Precisions were 0.982, 0.985, and 0.987; recalls were 0.935, 0.917, and 0.888, and F-measures for the 900 validation questions were 0.958, 0.950, and 0.935 for the thresholds of confidence rate of 50%, 60%, and 70%, respectively. The results did not change significantly according to the expertise of the authors of the validation questions for Kampo medicines (Supplementary Fig. 1). The higher the threshold of confidence rate was, the higher was the precision, and the lower were the recall and F-measure; but the differences were not significant.

The results of the system are shown in Fig. 3 and Supplementary Fig. 2. The total number of questions judged as TP and TN were approximately 90% of the 900 validation questions, regardless of the threshold. This indicates that the system gave the correct answers to the validation questions with high probability. The percentage of questions for which the system gave FN increased with increasing threshold of confidence rate (Fig. 2 and Supplementary Fig. 3).

## Discussion

We developed a novel drug information provision system for Kampo medicines using NLP. The developed system showed high performance with precision, recall, and F-measures of approximately 0.9 for all questions created by pharmacists regardless of their level of expertise in Kampo medicines. This NLC-based chatbot system is considered usable by pharmacists with any level of knowledge and experience because of its superior performance.

Although Google search is the information search tool most frequently used by medical students [32] and is also widely used by medical professionals, a study comparing the number of correct answers to questions about drug information found by Google search and by Micromedex (a database of drug and addiction information) found that the group using Micromedex had a significantly higher number of valid answers than the group using Google [20]. In 2018, Micromedex was incorporated into IBM Watson® [33]. Watson® has subsequently been shown to be useful in clinical settings as a chatbot system that provides extensive drug information [19, 20], although it has not previously been used to provide drug information in Japanese. Therefore, our system uses IBM Watson®. Other chatbot systems have used DialogFlow [34] (developed by Google) and Rasa [35] (developed by Rasa Technologies GmbH), but healthcare-related research with these two systems has focused on systems targeting patients [36–40] or on improving communication skills of medical professionals [40].

None of the previous studies that used IBM Watson® to develop a system to provide drug information validated the resulting system [19, 20]. In studies that used a similar application programming interface to predict diagnostic results using English medical record information, the accuracy of the predicted need for venography after musculoskeletal MRI examination was 0.9 [41], and the F value for breast-cancer-site prediction was 0.95 or higher [42]. The accuracy and F-values of this study were similar to these, although the studies cannot be directly compared.

Moreover, there have been no previous studies using the same Japanese language NLP used in this system. Previous studies using other Japanese language NLPs were meant to detect adverse reactions in electronic medical records, drug histories, articles, and social media [43–46]. The F-measures in these studies ranged from 0.65 to 0.90 [43–46], which is comparable to our findings.

The system worked well at the threshold value of 61% based on the ROC curve, with acceptably high levels of precision, recall, and F-measure. If we increased the threshold of the confidence rate, the system answered questions with “I do not have an answer.” This increased the number of FNs and decreased the recall because FN is included in the denominator. Contrarily, the precision values tended to become higher with the higher threshold of confidence rate because FP included in the denominator was decreased. These differences were, however, not significant.

The two main reasons that cause the system to give incorrect results even for the validation questions with correct answers are (Table 2) problems with structure of the NLC® and problems with the validation questions. Constitutionally, it is difficult for NLC® to distinguish between linguistically similar questions, for three reasons: (1) misclassification by NLC® because of confusing words (Category-sorting NLC® misclassification (15%) and Category NLC® misclassification (85%), no difference in the proportion of misclassifications among the six different Category NLC®s.); (2) a validation question covering more than one category or answers, such as concomitant use of Kampo formulas; and (3) a validation question with negative sentences (e.g., a validation question asked about “adverse events other than pseudoaldosteronism” but the system answered on “pseudoaldosteronism” because the NLC® assumes that a validation question is an affirmative sentence even though the question is written as a negative sentence.)

Additionally, ambiguous or indirect wording of a validation question may lead to a wrong answer including FN or FP depending on the threshold of the confidence rate. For example, the validation question, “Can I concomitantly use Hochuekkito in patients who are taking antihypertensive agents?” or “How many grams of licorice can I take in a day when I take Yokukansan?” did not lead to a correct answer because there is no yes/no answer or such direct description on the official information we referred to in the present study.

**Table 2:** Reasons that cause the validation questions with correct answers listed in the system to lead to incorrect answers

Classification			Reason for incorrect answer	Example of validation question (Original text was Japanese)	System answer	Answers by question authors
1	Problem with structure of NLC	1–1	Misclassification by NLC® (Category-sorting NLC® misclassification)	I want to know about patients to whom Yokukansan should be administered carefully. 抑肝散服用の際、慎重に投与すべき患者について知りたい。	Carefully administer Kampo formulas to patients with cardiovascular disorders (An answer in a different category NLC® with the answer by question author)	Careful administration of Yokukansan
			Misclassification by NLC® (Category NLC® misclassification)	In what patients is Shakuyakukanzoto contraindicated? 芍薬甘草湯の禁忌はどんな患者さんですか?	Indication of Shakuyakukanzoto (An answer in the same category NLC® with the answer by question author)	Contraindications to Shakuyakukanzoto
		1–2	Covering more than one category or answers (Concomitant drug)	Is it okay to take Hochuekkito and Shakuyakukanzoto together? 補中益気湯と芍薬甘草湯との飲み合わせは?	I do not have an answer. ^*^	Drug Interactions of Shakuyakukanzoto
		1–3	Question text structure	What side effects does Hochuekkito have other than pseudoaldosteronism? 補中益気湯は偽アルドステロン症以外に他にどのような副作用がありますか?	Contents about pseudoaldosteronism of Hochuekkito	Adverse reactions of Hochuekkito
2	Problem with validation question	2 − 1	Ambiguous question	Can I use Yokukansan for patients with gastrointestinal upset? 胃腸の調子が良くない患者さんに抑肝散を使ってもいいですか?	I do not have an answer. ^*^	Careful administration of Yokukansan
		2–2	Questions about content not mentioned in the package insert	Can Hochuekkito be used with patients taking antihypertensive drugs? 降圧剤を服用している患者さんに補中益気湯を併用してもよいですか?	Description of Hochuekkito	Drug Interactions of Hochuekkito

When the threshold of confidence rate is set to 61%

NLC® Natural Language classifier®

The process inside the NLC® is a black box; hence, similar questions could not be fixed to lead to correct answers. Watson® has knowledge originally trained by IBM and then trained further with data we provided. Because the learning model is created by integrating those data, the user cannot completely control Watson®’s behavior. As an example, consider “Misclassification by NLC®”: even if two questions and answers are learned that are distinguishable from our point of view, there is a possibility that Watson® will interpret them as identical questions and answers with the same meaning. To solve this problem, we are currently developing another artificial intelligence engine that enables us to expand the corpus and control. We hope to use the corpus created in this study to evaluate the usefulness of the newly developed QA system. Our data set is also available from the authors upon reasonable request so that other researchers can evaluate other QA systems.

Some validation questions and answers covered more than one category or Kampo formula name; the information on the two medications was in different categories. NLC® cannot classify questions, and it cannot answer questions about information outside the system if it is asked as a closed question. To solve this problem, we plan to adopt a keyword matching system that searches for keywords in the question text; if the keywords are included in the text, the system will display responses tied to predefined keywords. By including a keyword matching system, it will be possible to display the list of indications based on the keyword “indications” and treat the query as an open question on indications for the Kampo formula. Question text structure and ambiguous questions are difficult even for experts to answer. Thus, there are limitations to answering these questions using an NLC®-based system.

This study had several limitations that should be addressed in future studies. First, the system we developed can only answer registered questions. The system administrator must add new questions and answers whenever new questions come in. As with information retrieval on the Internet, prior studies have shown that it is necessary for application users to have the ability to judge whether the application is providing the correct information [20, 47]. Our system is mostly based on official public information. Second, the system should be used and further evaluated in a clinical setting (even though the validation questions were created based on questions that are usually asked in such a setting). We recruited pharmacists with various levels of expertise in Kampo medicines to create the validation questions. We expanded the number of validation questions from each participant to 100, but it is still necessary to collect validation questions from more pharmacists to validate the system. We also would like to confirm whether the system is useful for other healthcare specialists, such as doctors and nurses [48].

## Conclusions

We developed a novel drug information provision system for Kampo medicines using NLP. The precision, recall, and F-measure were 0.986, 0.915, and 0.949, respectively. We developed a system that gives appropriate answers to questions using natural language, assuming a clinical situation. We plan to confirm the results in the clinical field in the future. Other sources, such as the classic textbooks, will also be included.

## Electronic supplementary material

Below is the link to the electronic supplementary material.

Supplementary Material 1

Supplementary Material 2

Supplementary Material 3

Supplementary Material 4

## Data Availability

The datasets generated and/or analyzed during the current study are not publicly available but are available from the corresponding author upon reasonable request.

## List of Abbreviations

FN: False negative
FP: False positive
NLC®: Natural language classifier®
NLP: Natural language processing
TN: True negative
TP: True positive
