# Supplementary figures and images for: Development of a novel drug information provision system for Kampo medicine using natural language processing technology
Source: BMC Med Inform Decis Mak. 2023 Jul 13;23:119. doi: 10.1186/s12911-023-02230-3 (PMC10347708; doi:10.1186/s12911-023-02230-3)

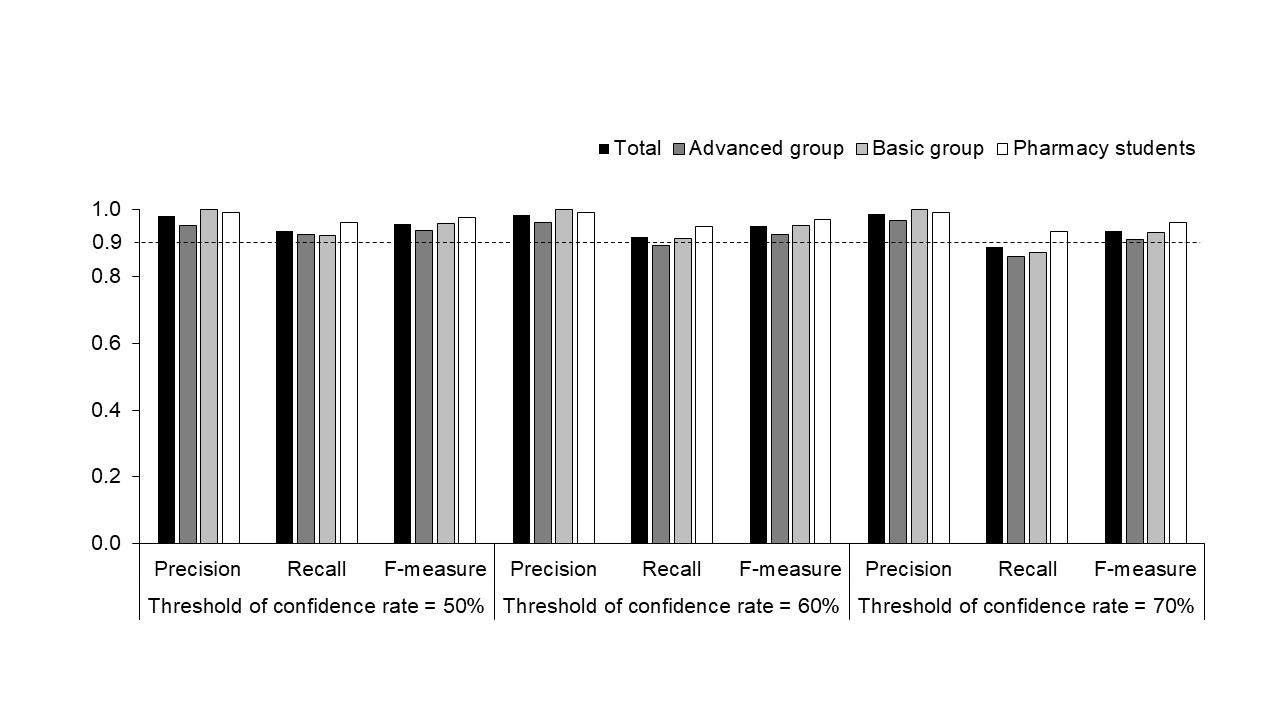

Supplement: Supplementary file 1 — Supplementary Material 1 [file 12911_2023_2230_MOESM1_ESM.jpg]

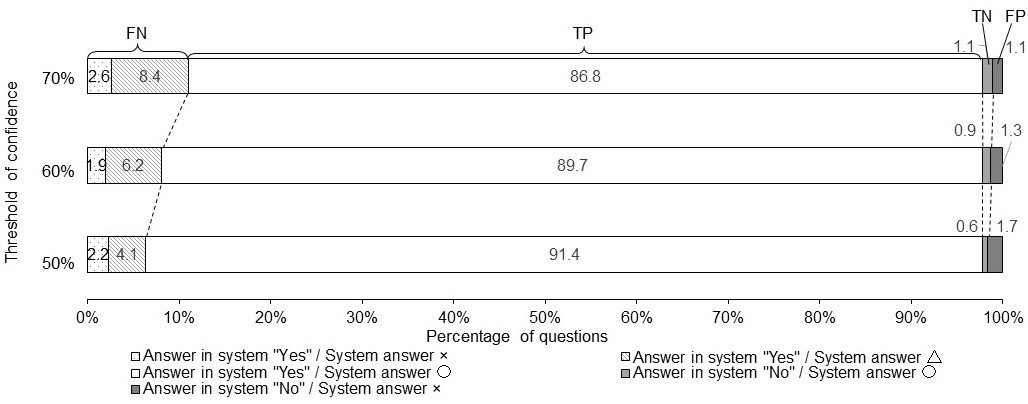

Supplement: Supplementary file 2 — Supplementary Material 2 [file 12911_2023_2230_MOESM2_ESM.jpg]

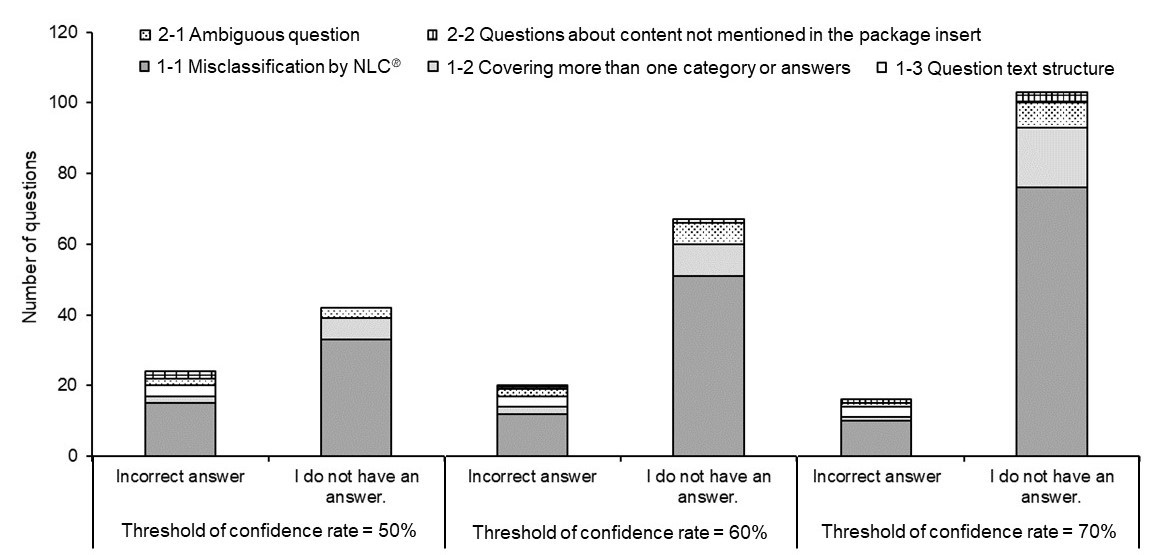

Supplement: Supplementary file 3 — Supplementary Material 3 [file 12911_2023_2230_MOESM3_ESM.jpg]
