# Supplementary material for: Development of a novel drug information provision system for Kampo medicine using natural language processing technology
Source: BMC Med Inform Decis Mak. 2023 Jul 13;23:119. doi: 10.1186/s12911-023-02230-3 (PMC10347708; doi:10.1186/s12911-023-02230-3)
Supplement: Supplementary file 4 — Supplementary Material 4 [file 12911_2023_2230_MOESM4_ESM.docx]

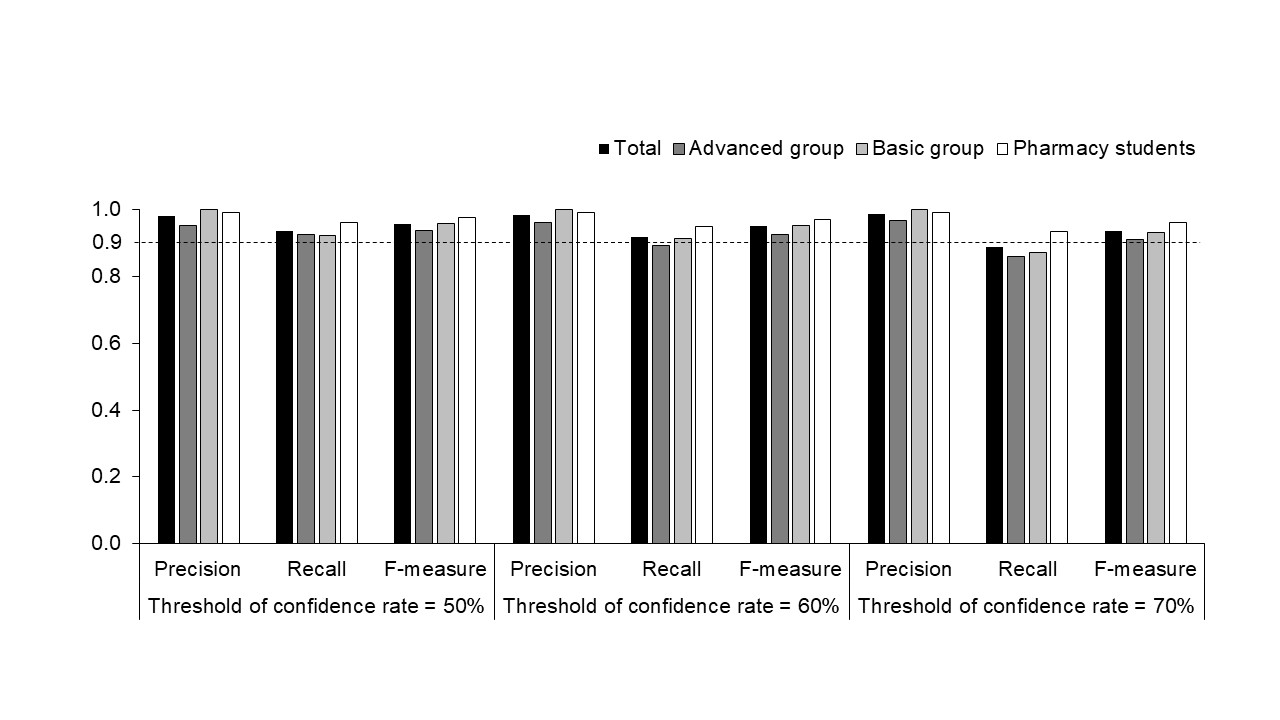


Supplementary Figure. 1 Precision, Recall, and F-measure by threshold of confidence rate

Advanced group Pharmacists with advanced knowledge and experience of Kampo medicines; Basic group Pharmacists with basic knowledge and experience of Kampo medicines


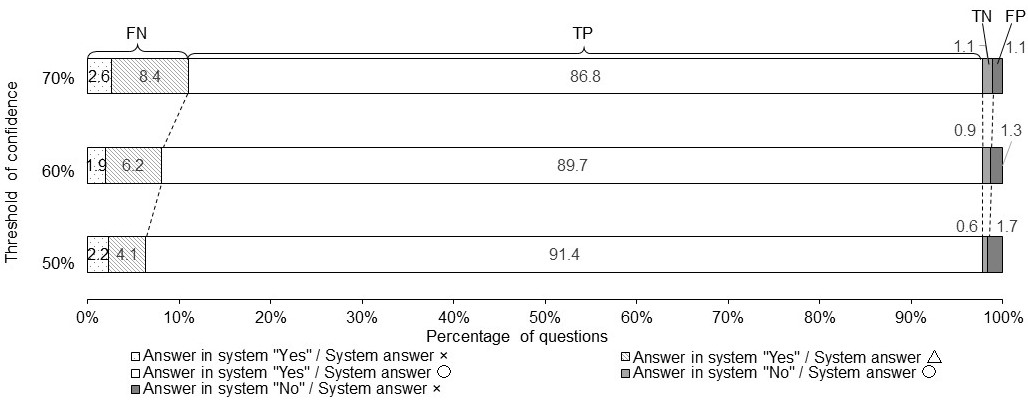


Supplementary Figure 2 The results of the system by the threshold of confidence rate

〇 means the system answers is the expected one. △ means the system answers were "I do not have an answer." × means the system answers were unexpected.

TP True positive; FN False negative; TN True negative; FN False negative


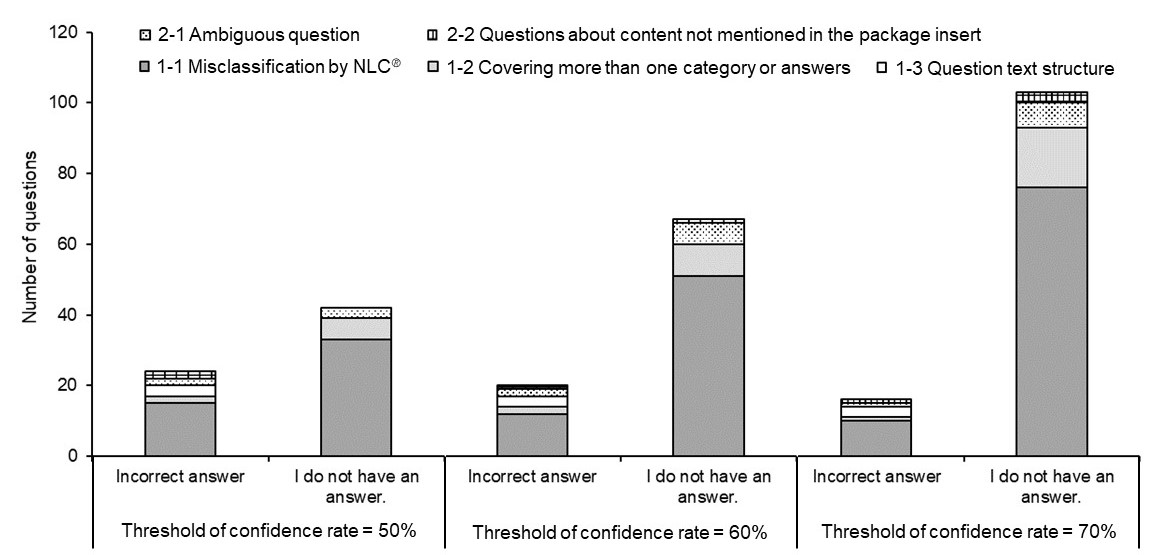


Supplementary Figure 3: Number of questions with correct answers listed in the system by threshold of confidence rate of why the question did not lead to the correct answer
